# Age-Related ^1^H NMR Characterization of Cerebrospinal Fluid in Newborn and Young Healthy Piglets

**DOI:** 10.1371/journal.pone.0157623

**Published:** 2016-07-08

**Authors:** Domenico Ventrella, Luca Laghi, Francesca Barone, Alberto Elmi, Noemi Romagnoli, Maria Laura Bacci

**Affiliations:** 1 Department of Veterinary Medical Sciences, University of Bologna, Bologna, Italy; 2 Centre of Foodomics, Department of Agro-Food Science and Technology, University of Bologna, Bologna, Italy; Imperial College London, UNITED KINGDOM

## Abstract

When it comes to neuroscience, pigs represent an important animal model due to their resemblance with humans’ brains for several patterns including anatomy and developmental stages. Cerebrospinal fluid (CSF) is a relatively easy-to-collect specimen that can provide important information about neurological health and function, proving its importance as both a diagnostic and biomedical monitoring tool. Consequently, it would be of high scientific interest and value to obtain more standard physiological information regarding its composition and dynamics for both swine pathology and the refinement of experimental protocols. Recently, proton nuclear magnetic resonance (^1^H NMR) spectroscopy has been applied in order to analyze the metabolomic profile of this biological fluid, and results showed the technique to be highly reproducible and reliable. The aim of the present study was to investigate in both qualitative and quantitative manner the composition of Cerebrospinal Fluid harvested form healthy newborn (5 days old-P5) and young (30-P30 and 50-P50 days old) piglets using ^1^H NMR Spectroscopy, and to analyze any possible difference in metabolites concentration between age groups, related to age and Blood-Brain-Barrier maturation. On each of the analyzed samples, 30 molecules could be observed above their limit of quantification, accounting for 95–98% of the total area of the spectra. The concentrations of adenine, tyrosine, leucine, valine, 3-hydroxyvalerate, 3-methyl-2-oxovalerate were found to decrease between P05 and P50, while the concentrations of glutamine, creatinine, methanol, trimethylamine and myo-inositol were found to increase. The P05-P30 comparison was also significant for glutamine, creatinine, adenine, tyrosine, leucine, valine, 3-hydroxyisovalerate, 3-methyl-2-oxovalerate, while for the P30-P50 comparison we found significant differences for glutamine, myo-inositol, leucine and trimethylamine. None of these molecules showed at P30 concentrations outside the P05 –P50 range.

## Introduction

Pigs represent an important animal model, being phylogenetically similar to primates [1], therefore extremely similar to humans, especially when compared to other models such as the murine one [2]. It is therefore necessary and mandatory to acquire as much knowledge as possible regarding porcine genetics and physiology in order to create specific models for each pathology and understand its correlation to its human analogue. When it comes to neuroscience, the porcine brain resembles the human brain in terms of weight, volume, cortical surface area, myelination, composition and electrical activity, and its development, just like in humans, extends from prenatal to early postnatal life [3]. Throughout the years, several porcine models carrying gene variants that cause neurological pathologies in men have been created [3] validating and proving the importance of this species in the laboratory and translational medicine.

Due to its position and fragility, Central Nervous System (CNS) samples can be hard to collect and the procedure may lead to severe damage, but Cerebrospinal fluid (CSF) represents a relatively easy to collect specimens that can provide important information about neurological health and function [4]. CSF functions include regulation of the intracranial pressure (ICP), regulation of the chemical environment of the CNS and intracerebral transport [5]. CSF is the product of plasma ultrafiltration and membrane secretion, usually clear and colorless [5]. It is nearly acellular, and does not contain erythrocytes in physiological conditions [6]. On average, dogs and cats have from 0 to 2 cells/μl, with specific normal nucleated cell count ranges for different species [7]. Protein concentration is usually very low: canine CSF samples usually show 10-40mg/dl of proteins compared to 5–7 g/dl in serum, the majority of which is represented by albumin (50–70%) [5]. Its production and absorption are the result of the interaction of several interfaces such as the Blood-Brain-Barrier (BBB) and the blood-CSF barrier [8], therefore it can vary depending on the age and the maturation of the above-mentioned barriers.

Recently, canine CSF small organic molecules profile, referred to as metabolome [9], was investigated using proton nuclear magnetic resonance (^1^H NMR) spectroscopy [10], in order to outline a fingerprint of healthy status useful for designing and interpreting clinical trials. ^1^H NMR is indeed ideally tailored for metabolomics investigations on biofluids, due to its high reproducibility, its intrinsic quantitative nature and the minimum sample preparation required [11]. Investigations of this kind have been, in the recent past, precious for characterizing diseases [12–13] and inflammation conditions [14]. In addition, focusing on rats, it was proven that CSF metabolomics can reveal changes in CNS metabolism in key conditions, strongly suggesting that interesting insights of CNS metabolism can be obtained also during animal growth [15]. In order for these investigations to be effective, a key role is covered by the exploration of the widest possible portions of the metabolome space [11], given by the number of quantified molecules and the by the knowledge about the connection between the metabolome profile and natural fluctuations of the physiological status, such as those connected to ageing.

Regarding the swine metabolome, the characterization of urine, serum, liver and kidney metabolome was recently performed, using both one and two-dimensional ^1^H and ^13^C nuclear magnetic resonance spectroscopy (NMR) and high-resolution magic angle spinning (HR-MAS) NMR [16]. The study provided valuable information for translational medicine, validating once again the importance of metabolomics.

The aim of the present study was to investigate in both qualitative and quantitative manner the composition of Cerebrospinal Fluid harvested form healthy newborn (5 days old) and young (30 and 50 days old) piglets using ^1^H NMR Spectroscopy, and to analyze any possible difference in metabolites concentration between age groups, related to age and Blood-Brain-Barrier maturation.

## Material and Methods

### Animals

Animals used in this study were Large White x Landrace x Duroc commercial hybrids. The total amount of animals sampled for this study was 44: 17 5-days old piglets (P05), 18 30-days old piglets (P30) and 9 50-days old piglets (P50). None of the animals was sampled at two different time points. Pregnant sows (for P05 animals) and weaned piglets (for P30 and P50 animals) were delivered to our facility from the same farm (Societa' Agricola Pasotti S.s, Imola 40026, Italy) in order to obtain a population as consistent and coherent as possible. P05 animals were housed with the sow in the farrowing crate with a heating lamp, while P30 and P50 in multiple pens according to their age. Weaned animals (P30-P50) were fed an age-appropriate commercial diet twice a day. All animals were enrolled as negative controls or as pre-treatment individuals in different protocols approved by the Italian Ministry of Health (art.7, D.Lgs 116/92), and were monitored at least once a day by the veterinarian. The sampling procedure was performed under general anaesthesia in order to avoid stress and guarantee the welfare of the animals. All pigs were constantly monitored during and after the procedure to rule out any possible complication. According to the individuals’ protocols, all animals were eventually euthanized upon intravenous administration of Tanax (embutramide, mebenzonium iodide and tetracaine hydrochloride; 0.3 ml/kg; MSD Animal health, Milano, Italy) after general anesthesia.

### Sampling procedure

Animals were considered to be healthy on the basis of clinical examination and blood tests, including a Complete Blood Count (CBC) and Chemistry Profile. Sampling procedures were performed as previously described by Romagnoli et al. [17]. Briefly, animals were anesthetized using inhalational induction with 8% Sevoflurane (SevoFlo; Abbott Laboratories, Chicago, IL, USA) in a oxygen and air mixture (1:1). After endotracheal intubation, piglets were positioned in lateral recumbency, and the dorsal area of the neck was clipped and surgically prepared. Cisterna Magna was punctured using a 75mm 22gauge spinal needle, and 1 ml of clear, non-hemorrhagic Cerebrospinal Fluid was collected into a sterile cryogenic tube and immediately frozen in liquid nitrogen, then stored in a-80°C freezer until analysis.

### NMR spectra acquisition and treatment

The samples constituting each of the three groups were collected in two batches of similar size. The samples from each batch were prepared for ^1^H-NMR analysis simultaneously, to minimize possible variability due to preparation conditions. To meet the sample volume specifications of the NMR probe, 300 μl of CSF were added to 300 μl of distilled water. The samples were centrifuged for 15 minutes at 15,000 rpm at 4°C. 500 μl of supernatant were added to 100 μl of a D_2_O 1M phosphate buffer at pH 7.00 solution of 3-(trimethylsilyl)-propionic-2,2,3,3-d4 acid sodium salt (TSP) 6.25 mM, added as reference compound, and of 2 mM sodium azide, to avoid bacteria proliferation [18]. To minimize time at room temperature between sample preparation and spectra acquisition, the samples were stored at -20°C prior to analysis for a time varying between 12 and 24 hours. Immediately before spectra acquisition the samples were thawed and centrifuged again. The samples underwent analysis in random order, requiring a maximum of 6 hours. ^1^H-NMR spectra were recorded in 5 mm NMR tubes at 298 K with an AVANCE III spectrometer (Bruker, Milan, Italy) operating at 600.13 MHz.

Following Öhman et al. [19], the signals from broad resonances originating from large molecules were suppressed by a CPMG-filter composed by 400 echoes with a τ of 400 μs and a 180° pulse of 24 μs, for a total filter of 330 ms. The HOD residual signal was suppressed by means of presaturation. This was done by employing the cpmgpr1d sequence, part of the standard pulse sequence library. Each spectrum was acquired by summing up 256 transients using 32 K data points over a 7184 Hz spectral window, with an acquisition time of 2.28s. In order to apply NMR as a quantitative technique [20], the recycle delay was set to 5s, keeping into consideration the relaxation time of the protons under investigation. Pre-analytical sample management protocol and NMR experiments are conveniently summarized in S1 File, according to Rubtsov et al. guidelines [21]. The signals were assigned by comparing their chemical shift and multiplicity with Chenomx software (Chenomx Inc., Canada, ver 8.1) standard (ver. 10) and HMDB (ver. 2) data banks, as described in detail in S1A and S1B Fig. In case of ambiguity, proton-proton 2D experiments were performed, as shown in S1C Fig.

### Data analysis

Spectra were manually phase adjusted by means of Tospin (ver 3 –Bruker, Milan, Italy) and then transferred to Mestrenova (ver 10.0.2—Mestrelab Research S.L., Spain). Here a line broadening of 0.3 Hz was applied and an alignment towards TSP signal, set to 0 ppm, was applied. The baseline was adjusted by means of the Whittaker smoother algorithm [22], by applying a filter of 100 and a smoothing factor of 16384. Finally, the irregularities of the magnetic field leading to imperfections of the signals shape were compensated by reference deconvolution, by considering TSP singlet and a target linewidth of 1.2 Hz. No manual alignment of the signals was necessary, different to other body fluids [23]. Differences in water content among samples were taken into consideration by probabilistic quotient normalization [24], applied to the entire spectra array.

Data analyses were performed on R environment (version 3.2.2; the R Foundation for Statistical Computing, Vienna, Austria). Molecules showing different concentrations between time points were analyzed using a non-parametric Mann-Whitney U test. A probability lower than 0.05 was considered as significant, adjusted for multiple comparisons through Bonferroni correction.

Models of discriminant analysis based on projection on latent structures (PLS-DA) were built and graphically represented by means of the package mixOmics, formerly known as integrOmics [25]. For the purpose 75% of the samples from each group were randomly employed as a training set, while the remaining samples were used to test the model’s performance. The optimal number of new space components was found by 10 fold cross-validation. The trends in the individuals distribution were highlighted by representing them in the XY-variate subspace described by PLS-DA model. For each component, the importance of the molecules in the samples distribution was highlighted by calculating the correlation between each metabolite and the selected latent variable, thus obtaining the so called correlation circle plot. To rank the overall importance of each molecule in the model, we calculated its variable importance over projection (VIP) [26]. As an alternative criterion, PLS-DA models were built in their sparse version (sPLS-DA) [27]. Briefly, sPLS-DA algorithm does not build DA models on the entire set of molecules, but pre-selects only those with the highest discriminative power, thus indirectly acting as a molecules ranking procedure. In our case, for each iteration models of increasing complexity were built by adding one new molecule at each iteration to a starting number of two.

The concentrations of the molecules observed in the present work spanned four orders of magnitude. The most concentrated molecules, with no biological reasons, would have dominated any multivariate model if employed as is. This forced us to scale each concentration to unit variance. This choice reduced the possibility for the reader to visually rank the molecules according to their importance in the models. Such drawback was solved by setting up a cascade analysis protocol, where each multivariate algorithm refined the information granted by the previous.

## Results

The sampling procedure proved to be strong and reliable, allowing the operator to collect blood-contamination free samples, suitable for analysis. Moreover, none of the animals showed alterations related to the procedure.

All the raw data are showed in S1 Table.

**Fig 1:**
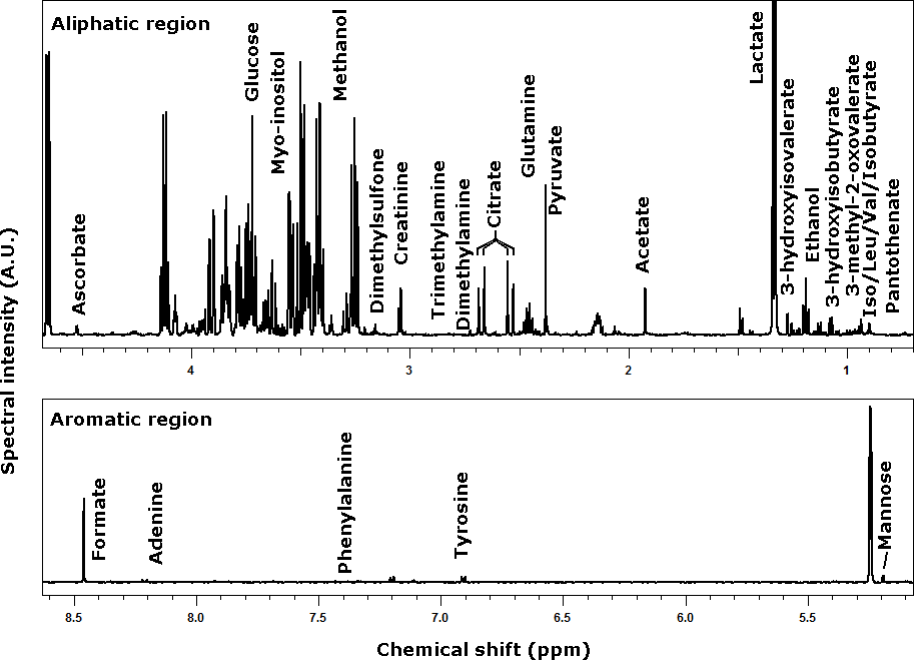
A 1D-NMR spectrum of CSF from a 30d pig, representative of all the registered spectra.

A 1D-NMR spectrum of CSF from a 30d pig, representative of all the spectra registered in the present work, is depicted in Fig 1. On each of the analyzed samples, 29 molecules could be observed above their limit of quantification, accounting for 95–98% of the total area of the spectra. Their concentration was obtained by integrating each spectrum over the ranges listed in Table 1, comprising complete multiplets, as in the case of lactate, or portions, as in the case of glucose. The concentration of the molecules quantified by NMR in CSF are reported in Table 2.

**Table 1:** Location of the NMR signals employed for molecules quantification, identified in CSF of pigs in the 5–50 days range

Molecule	Range*	Protons**	Molecule	Range*	Protons**
Glucose	3.698–3.711	0.25	3-hydroxyisobutyrate	1.075–1.085	1.5
Lactate	1.298–1.356	3	Adenine	8.193–8.226	1
Formate	8.444–8.473	1	Acetate	1.914–1.933	3
Glutamine	2.433–2.484	2	Tyrosine	6.891–6.923	2
Citrate	2.521–2.563	2	Leucine	0.951–0.98	3
myo-inositol	3.608–3.636	1.5	Pantothenate	0.877–0.882	3
Pyruvate	2.373–2.385	3	Phenylalanine	7.416–7.452	2
Ascorbate	4.515–4.527	1	Valine	1.035–1.056	3
Ethanol	1.171–1.206	3	3-hydroxyisovalerate	1.267–1.277	6
Mannose	5.185–5.197	0.5	Dimethylamine	2.728–2.718	6
Creatinine	3.046–3.056	3	Isoleucine	1.004–1.023	3
Alanine	1.472–1.497	3	Isobutyrate	1.057–1.065	3
Creatine	3.036–3.045	3	Trimethylamine	2.879–2.894	9
Dimethyl sulfone	3.154–3.161	6	3-methyl-2-oxovalerate	1.117–1.136	3
Methanol	3.362–3.37	3			

* Portion of spectrum (expressed in ppm) where the multiplet employed for quantification were identified

** Number of protons giving rise to the multiplet. A fractional number indicates that a multiplet was not considered in its entirety.

**Table 2:** Concentration of the molecules quantified by NMR in CSF (mM).

	P05*	P30	P50	P05—P50 P-values
**Lactate**	2.42E+00 ± 3.68E-01	2.11E+00 ± 2.69E-01	2.84E+00 ± 3.99E-01	2.14E-02
**Glucose**	2.01E+00 ± 1.89E-01	1.72E+00 ± 2.89E-01	1.92E+00 ± 3.89E-01	8.33E-01
**myo-inositol**	3.87E-01 ± 7.38E-02	3.86E-01 ± 1.18E-01	6.03E-01 ± 1.49E-01	1.23E-03**
**Ethanol**	3.76E-01 ± 4.85E-01	8.42E-01 ± 1.51E+00	1.45E-01 ± 1.53E-01	2.87E-01
**Glutamine**	2.78E-01 ± 1.81E-02	3.56E-01 ± 9.99E-02	5.14E-01 ± 1.19E-01	6.40E-07**
**Formate**	2.27E-01 ± 2.82E-02	1.78E-01 ± 5.03E-02	2.56E-01 ± 4.71E-02	1.20E-01
**Citrate**	1.99E-01 ± 3.10E-02	1.27E-01 ± 2.19E-02	2.30E-01 ± 4.85E-02	1.33E-01
**Pyruvate**	1.52E-01 ± 3.26E-02	1.46E-01 ± 2.47E-02	1.13E-01 ± 2.02E-02	2.86E-03
**Ascorbate**	1.06E-01 ± 5.17E-02	1.11E-01 ± 6.94E-02	7.30E-02 ± 1.00E-02	3.96E-01
**Mannose**	9.16E-02 ± 1.09E-02	9.05E-02 ± 2.13E-02	7.79E-02 ± 1.51E-02	5.10E-02
**Alanine**	7.53E-02 ± 1.47E-02	9.52E-02 ± 1.61E-02	5.74E-02 ± 1.31E-02	1.10E-02
**Acetate**	6.72E-02 ± 2.30E-02	4.93E-02 ± 3.56E-02	6.96E-02 ± 1.88E-02	7.11E-01
**Creatine**	5.28E-02 ± 1.09E-02	4.38E-02 ± 7.42E-03	5.54E-02 ± 1.44E-02	7.11E-01
**Creatinine**	3.54E-02 ± 3.40E-03	4.39E-02 ± 6.01E-03	4.99E-02 ± 7.82E-03	2.56E-06**
**3-hydroxyisobutyrate**	3.04E-02 ± 7.63E-03	1.63E-02 ± 9.46E-03	2.22E-02 ± 4.03E-03	5.27E-03
**Leucine**	2.88E-02 ± 4.40E-03	2.11E-02 ± 3.87E-03	1.61E-02 ± 3.02E-03	6.40E-07**
**Adenine**	2.73E-02 ± 8.21E-03	1.47E-02 ± 7.44E-03	1.60E-02 ± 6.74E-03	4.09E-04**
**Tyrosine**	2.71E-02 ± 7.18E-03	1.19E-02 ± 3.75E-03	8.58E-03 ± 1.53E-03	6.40E-07**
**3-methyl-2-oxovalerate**	2.21E-02 ± 4.52E-03	1.36E-02 ± 5.88E-03	1.40E-02 ± 3.79E-03	2.30E-04**
**Methanol**	1.44E-02 ± 1.89E-03	2.74E-02 ± 1.45E-02	4.08E-02 ± 1.77E-02	6.40E-07**
**3-hydroxyisovalerate**	1.22E-02 ± 3.35E-03	4.22E-03 ± 2.10E-03	3.90E-03 ± 9.29E-04	6.40E-07**
**Valine**	1.13E-02 ± 2.47E-03	8.10E-03 ± 3.37E-03	5.52E-03 ± 1.85E-03	6.40E-07**
**Phenylalanine**	8.71E-03 ± 2.58E-03	7.56E-03 ± 2.38E-03	6.23E-03 ± 2.00E-03	2.50E-02
**Dimethyl sulfone**	8.45E-03 ± 2.09E-03	1.05E-02 ± 4.88E-03	5.35E-03 ± 2.08E-03	2.86E-03
**Isoleucine**	5.48E-03 ± 1.54E-03	6.53E-03 ± 4.49E-03	3.97E-03 ± 1.46E-03	1.31E-02
**Pantothenate**	3.91E-03 ± 2.76E-03	4.94E-03 ± 4.16E-03	2.58E-03 ± 8.16E-04	3.39E-01
**Dimethylamine**	2.71E-03 ± 5.03E-04	2.79E-03 ± 5.84E-04	2.27E-03 ± 6.14E-04	9.52E-02
**Isobutyrate**	2.53E-03 ± 7.23E-04	2.06E-03 ± 1.46E-03	3.06E-03 ± 1.24E-03	3.12E-01
**Trimethylamine**	7.57E-04 ± 3.49E-04	4.56E-04 ± 4.29E-04	4.97E-03 ± 3.82E-03	9.02E-04**

*The concentrations are expressed as mean ± standard deviation. The molecules are sorted according to their concentration at P05.

** Molecules showing statistical differences between P05 and P30.

**Fig 2:**
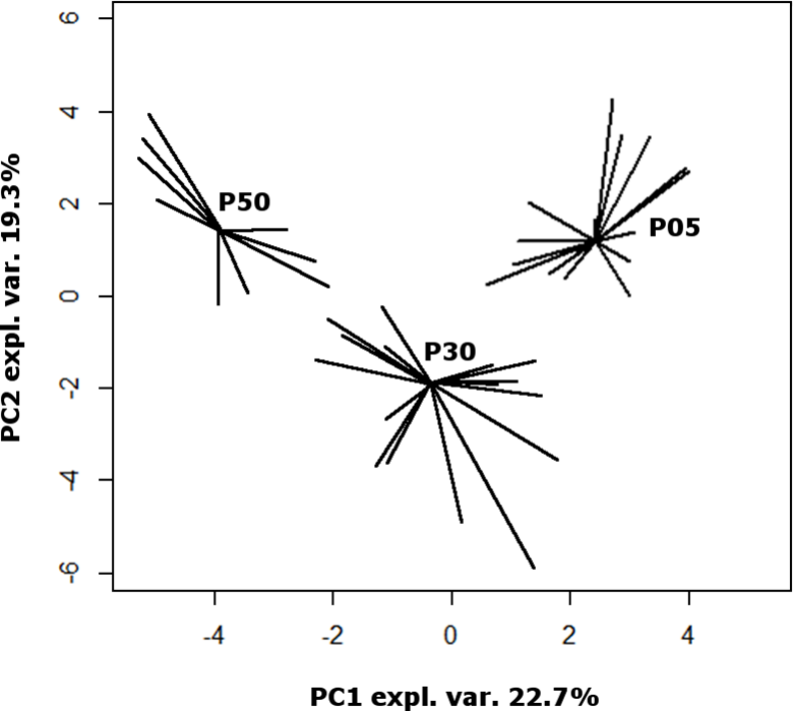
Scoreplot of a PCA model built on the concentrations of 29 molecules listed in Table 1. For each group, segments are drawn from each sample position to the median of the group. “Expl.Var.” stands for explained variance of the original data.

To gain an overall first impression of how the samples spread in the 29 dimensions space, for each P05 sample we calculated the median euclidean distances from the other P05 samples and from the samples collected at P50. The so obtained intergroup/intragroup distance ratio resulted statistically higher than 1 (P<7.63 E-6). The same significant difference was found for the P05 –P30 and the P30-P50 comparisons. In the same 29 dimension space, we found that P30 samples were equally distant from those collected at P05 and to those collected at P50. These observations show that the metabolome of each of three groups of samples was different from the others and that the characteristics of the samples at P30 were intermediate. To have a pictorial representation of this status, we calculated a PCA model on the centered and scaled concentrations of the molecules (Fig 2). The first principal component, even if representing the 22.7% of the total samples variance only, allowed a clear view of the samples metabolome overall evolution over time.

To focus on the molecules that mostly contributed to such overall trend along swine growth, we performed comparisons between P05 and P50 sample on a molecular basis. The concentration of adenine, tyrosine, leucine, valine, 3-hydroxyvalerate and 3-methyl-2-oxovalerate was found to decrease between P05 and P50, while the concentration of glutamine, creatinine, methanol, trimethylamine and myo-inositol was found to increase. The P05-P30 comparison was also significant for glutamine, creatinine, adenine, tyrosine, leucine, valine, 3-hydroxyisovalerate, 3-methyl-2-oxovalerate, while for the P30-P50 comparison we found significant differences for glutamine, myo-inositol, leucine and trimethylamine. None of these molecules showed at P30 concentrations outside the P05 –P50 range.

**Fig 3:**
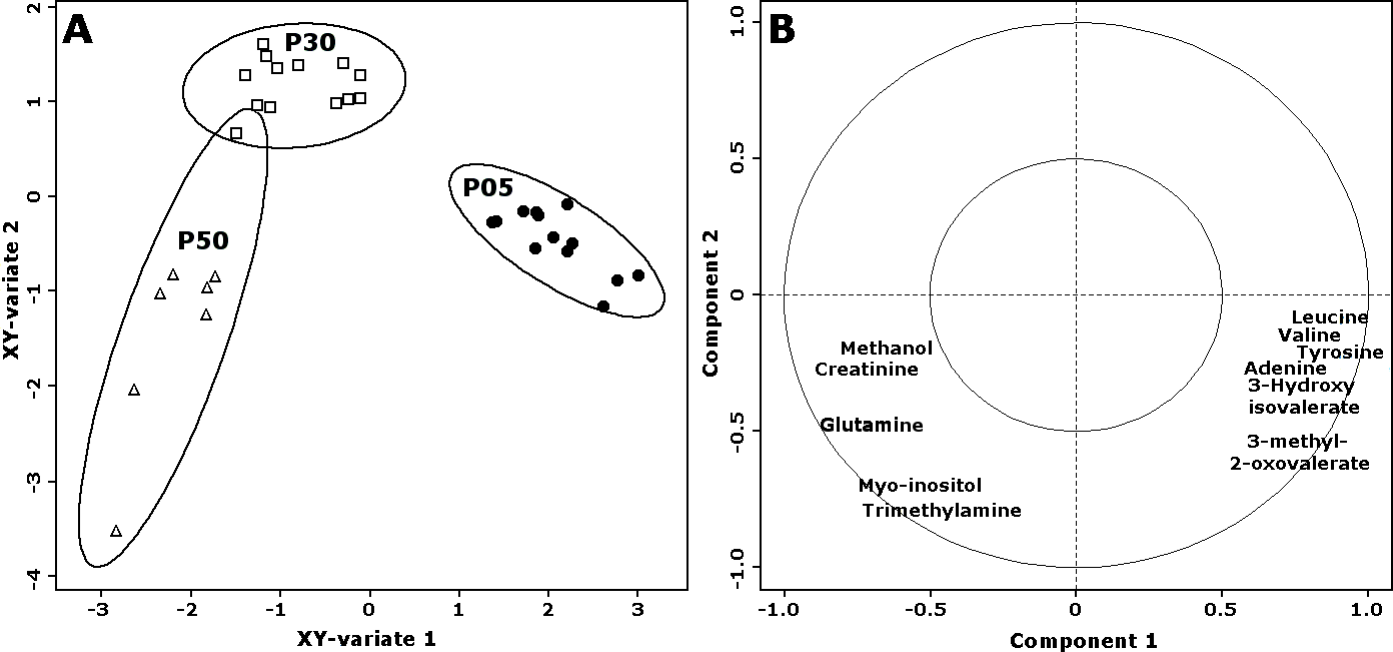
**Scoreplot (A) and correlation plot (B) of one of the PLS-DA models built.** The base of data was represented by the centered and scaled concentrations of the 11 molecules showing statistically significant differences between P05 and P50. The inner and outer circles of the correlation plots represent correlations of 0.5 and 1 respectively. P05, P30 and P50 samples are represented with filled circles, empty squares and empty tringles respectively.

Focusing on the molecules that were found to differ between P05 and P50 samples, we desired to robustly rank them according to their importance in discriminating the three groups of samples. For the purpose, we followed a double procedure based on PLS-DA with VIP calculation on one side, and on sPLS-DA models on the other side. In detail, the concentrations of the molecules were employed to build 100 PLS-DA models, one of which presented in Fig 3. The discriminant models were preferred to the regression counterparts because we did not have any a-priori information about linearity of metabolome evolution along swine growth. Over the 100 built models, the average variance of the original samples explained by the first component was 83.1% ± 1.7%. No error was made in the assignment of the samples constituting the test set. The average number of latent components required by the models for such correct classification was 2.25 ± 1.67, with a median of 1. Over the 100 models, tyrosine, 3-hydroxyisovalerate and trimethylamine were the only variables with average VIP above 1, which is typically considered as a safe threshold of importance for variables in PLS [26].

For each PLS-DA model, we created also a sPLS-DA counterpart, to take advantage of its pre-screening procedure of the molecules with the highest discriminating power. Over the 100 sPLS-DA models, the system selected as most important 3-hydroxyisovalerate and tyrosine 100 times, and creatinine, leucine and trimethylamine, 68, 61 and 57 times respectively.

## Discussion

In the present work, we wanted to characterize the metabolome of cerebrospinal fluid of healthy newborn piglets, and to observe its modifications over the very first stages of life. The metabolome characterization was conveniently performed by means of ^1^H-NMR, one of the leading techniques in the field due to its high reproducibility. The modifications along swine development were looked for through a multistep protocol, based on uni- and multivariate algorithms, able to put in progressive evidence the molecules mainly evolving over time.

The time points used in this paper were 5, 30 and 50 days of life. As previously stated, all the animals analyzed in this study were enrolled in different experimental protocols held in our facility, therefore samplings occurred according to the previously chosen times. One of the few attempts to compare swine and human ages suggests that our time points might approximately mimic 1, 6 and 12 months of age in humans. [28]. Due to the lack of specific data, every time point can provide important insights, and the 30 days old model has recently been used to describe new techniques [17, 29] and characterize new gene therapy patterns [30].

Before discussing the most representative molecules and the ones showing statistical differences, it is important to acknowledge the presence of ethanol within all of the samples. Its role in the CSF has recently been thoroughly analyzed by van der Sar et al. [31]: its presence has been interpreted as either a contaminant or a disease process-related molecule, but the same work also proved its capability to diffuse into ex vivo CSF samples via air transmission, therefore altering the metabolome post sample collection [31]. In addition, it is important to mention that the area of the neck used for puncture was prepped by means of a chlorhexidine gluconate/ethanol surgical scrub. Therefore, considering this potential contaminative source and the fact that our animals showed no signs of disease, its presence is most likely to be due to contamination.

The most representative molecules, consistently throughout the analyzed groups, are glucose and lactate, related to energy metabolism. This finding is coherent with the data already available for dogs [10] and humans [32]. Glucose represents the most important energy supply, and, within the CNS, plays an important role in the synthesis of pivotal neurotransmitters as glutamate, GABA and aspartate [33], so that high concentrations are highly common. Regarding the neonatal brain, the specific glucose metabolism has been recently described, showing that, compared to adult brains, more of this compound is prioritized to the pentose phosphate pathway (PPP). The latter is pivotal for the synthesis of DNA/RNA and the regeneration of NADPH [34]. Alongside, lactate represents the major product of anaerobic glycolysis, so that its high concentration is consistent with CSF composition of any animal species. As a diagnostic marker, high concentrations of lactate are reported to be related with both hypoxia and bacterial infections, particularly meningitis [35]. The characterization of glycolysis through the CSF metabolome might have extreme diagnostic capabilities. Indeed it seems that the shift from aerobic to anaerobic glycolysis is related with improvements of cognitive status in HIV affected patients [36].

Out of the 29 identified molecules, only 11 showed significant differences between the three groups, with P30 animals leaning more towards P5 or P50 depending on the specific compound. It has already been demonstrated that the concentration of the metabolites within CSF is influenced by several factors such as genetics, breeding, diet, and environment [37]. The impact of age on the CNS metabolome is nowadays an object of study, with one the most recent aiming to describe the brain metabolome of rats throughout their lifespan. The analyses proved that compared with regional differences, age contributed more substantially to the detected differences [38]. Differences in metabolites concentrations may be due to a number of factors related to both cerebral and systemic processes, including the maturation of the Blood-Brain-Barrier (BBB), age-related differences in brain metabolic rates and blood composition. It is therefore very likely to detect both increasing and decreasing trends when analyzing these molecules. Obviously, molecules fluctuating between individuals may be due as well to underlying pathologies. CSF metabolomics is indeed one of the most innovative and promising technique for the diagnosis of CNS diseases such as glioma [39] and leptomeningeal carcinomatosis [40]. Our animals were proved to be healthy on the bases on clinicopathological evaluations, and no individual differences were noticed. It is therefore safe to say the differences between homogenous groups are to be imputed to the progression of the developmental stages.

Glutamine (glutamatergic system), myo-inositol (second messenger pathways), creatinine (energy metabolism), methanol (diet/systemic metabolism), and trimethylamine (diet/systemic metabolism) were found to significantly increase in concentration over time. The first three molecules, while ubiquitous, are involved in brain metabolism processes [10]; their increasing trend might reflect higher cerebral activity rates alongside with higher blood concentrations and BBB permeability. It is important to discuss the trend of glutamine, since it has been proven that it can act as an important biomarker in human medicine: an increase in its CSF concentration can be related to pathological processes such as depression [41]. On the other hand, concentrations lower than normal were detected in patients affected by multiple sclerosis [42]. We suggest that its increase in our animals may be reported to a higher rate in glucose metabolism and the proliferation of astrocytes within the neonatal brain. Astrocytes are the only cells containing Glutamine Synthetase, the only enzyme capable of converting glutamate and ammonia to glutamine in the mammalian brain [43]. The number of astrocytes at birth is sensibly lower when compared to adults, but the majority of gliogenesis occurs during the first weeks of life, making them the most present cells of CNS [44]. These cells play a pivotal role in the function and maintenance of the BBB [45], therefore, if we relate high concentrations of glutamine to an increasing number of astrocytes, we might use this finding as an indirect index of BBB maturation. Methanol and Trimethylamine, on the other hand, are considered to be waste products of food and/or systemic metabolism. In particular, metabolic methanol may occur as a result of fermentation by gut bacteria and metabolic processes involving S-adenosyl methionine [46]. Food probably represents the most important source of exogenous methanol and trimethylamine, and it is important to state that animals enrolled in the three groups, received different feed. P05 did not receive any solid feed in addition to the milk produced by the sows. P30 animals were freshly weaned (28^th^ day of life) piglets that had just started eating solid feed, and P50 had eaten solid feed for the previous 20 days. This feeding difference may be related to higher methanol and trimethylamine blood concentrations reflecting in increasing CSF concentrations. Unfortunately, due to the complexity of the metabolic pathways, partly still unknown, it is not possible to rule out any other hypothesis regarding these molecules. However it is important to stress this important correlation between metabolomics and food. The reflection of ingested molecules and their metabolites in the CSF metabolome profile might open new doors for more in depth analyses. This statement is true especially when it comes to orally administered integrators expressing their beneficial effects on the CNS such as quercetin [47] or to potential toxic agents introduced with food such as mercury [48].

Molecules showing significant decrease were adenine (protein synthesis/cellular respiration), tyrosine, leucine, valine (amino acids),and 3-hydroxyisovalerate and 3-methyl-2-oxovalerate (amino acid metabolism). It is very well acquainted that amino acids take part in a variety of biological processes, regulating in general the proteome. Free amino acids are pivotal for protein synthesis, acting as substrate for the growth and maintenance of tissues. They have impact on several events such as gene expression or transcription, immune response and autophagy. Moreover, as signaling molecules, they might have regulatory functions on protein turnover [49]. The influx of amino acids from the blood to the brain, and the regulatory role of the BBB has been extensively reviewed by Saunders et al [50], stating that essential amino acids are transported into the brain to a greater extent than non-essential ones; the correlation with BBB developmental state was not investigated. 3-Hydroxyisovalerate is derived from isovaleryl-CoA, a catabolic intermediate of leucine, and its concentration in adults’ CSF was analyzed in the human metabolome database [32]. It was suggested that a decrease of 3-hydroxyisovalerate level in the human serum may be related to the development of neoplastic disease and in particular pancreatic cancer [51], but its role in the CSF has not been investigated. 3-methyl-2-oxovalerate, just like the previously mentioned compound, is involved in amino acid metabolism, representing the first degradation product of isoleucine [52]. In authors’ opinion, the decrease of nucleobases and amino-acids, and of their metabolism products, might be related with the maturation and the increase in selectivity of the blood-brain barrier, but it is not possible to exclude an increase in their utilization within the CNS, thus making free concentrations lower. The concentrations of amino-acids in infant and newborn CSF were analyzed due to their possible role as diagnostic biomarkers for inborn errors of metabolism [53], therefore standard reference for piglets can be of extreme interest, especially considering transgenic models for this class of diseases.

Alongside the physiological analysis of CSF, a secondary aim of the present paper was to evaluate the evolution and the dynamics of the blood brain barrier in the swine model. Our data doesn’t allow us to hypothesize a physiological age range for complete maturation of the blood brain barrier as suggested by differences between 30 and 50 days old piglets. Further studies on older animals are needed in order to be able to set a possible mark on complete maturation when no differences will be noticed between two age groups.

In conclusion, the present paper seems to supply with robust and valuable data regarding the physiological description of the swine CSF, providing new knowledge about such an important animal model. The extensive statistical analyses proved the 3 groups to be well assorted and homogeneous, providing more relevance and impact to the results and interesting hints for further studies about the BBB physiology. Diagnostic procedures involving CSF analyses for the swine medicine itself are very unlikely to be performed on a routine basis, but the situation is completely different regarding pigs enrolled in translational medicine protocols. This approach towards the quali-quantitative analysis of CSF and the maturation of the BBB is indeed an important step for the refinement and the standardization of the swine model.

## Supporting Information

S1 Fig Molecules assignment. (DOCX)

S1 File NMR additional info. (DOCX)

S1 Table Raw data. (XLSX)
