# Supplementary material for: Age-Related 1H NMR Characterization of Cerebrospinal Fluid in Newborn and Young Healthy Piglets
Source: PLoS One. 2016 Jul 8;11(7):e0157623. doi: 10.1371/journal.pone.0157623 (PMC4938496; doi:10.1371/journal.pone.0157623)

**Fig. a.** Pictorial representation of molecules quantification procedure by ^1^H-NMR. For each molecule listed in table 1 of the main document, a convenient portion of each of the registered spectra is shown. The dashed line highlights the extremes of the signals reported in table 1.


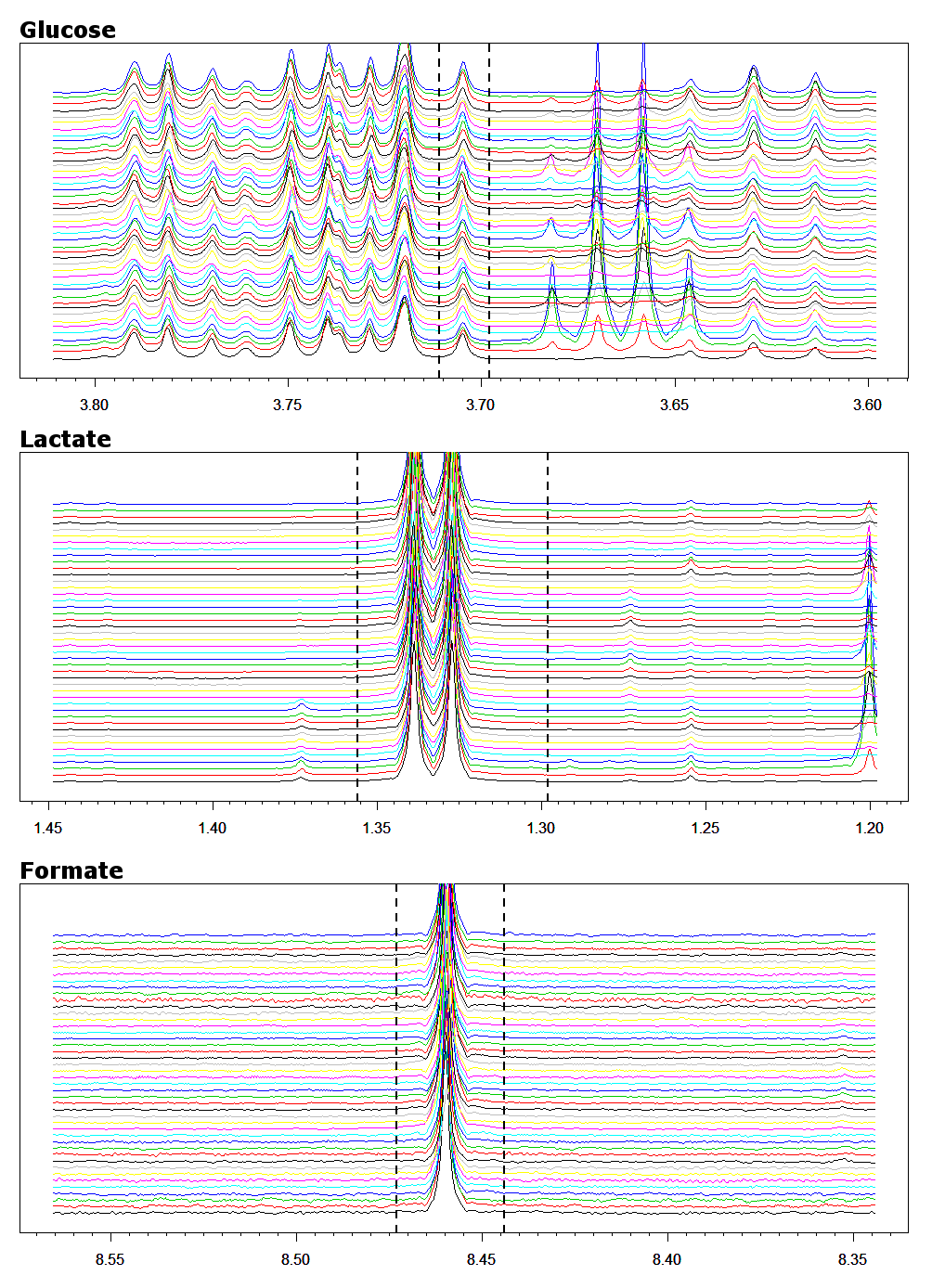


Figure continues from previous page


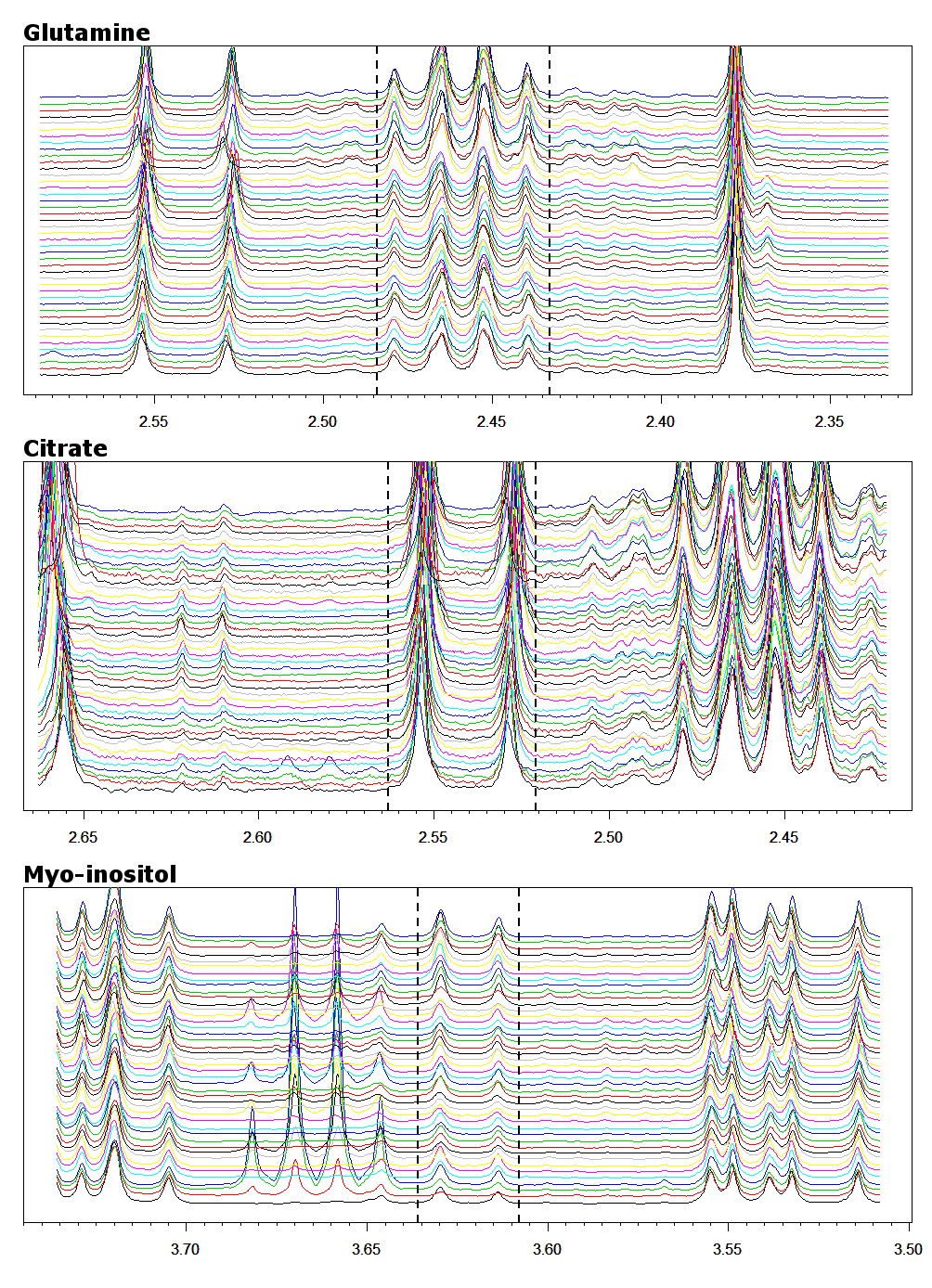


Figure continues from previous page


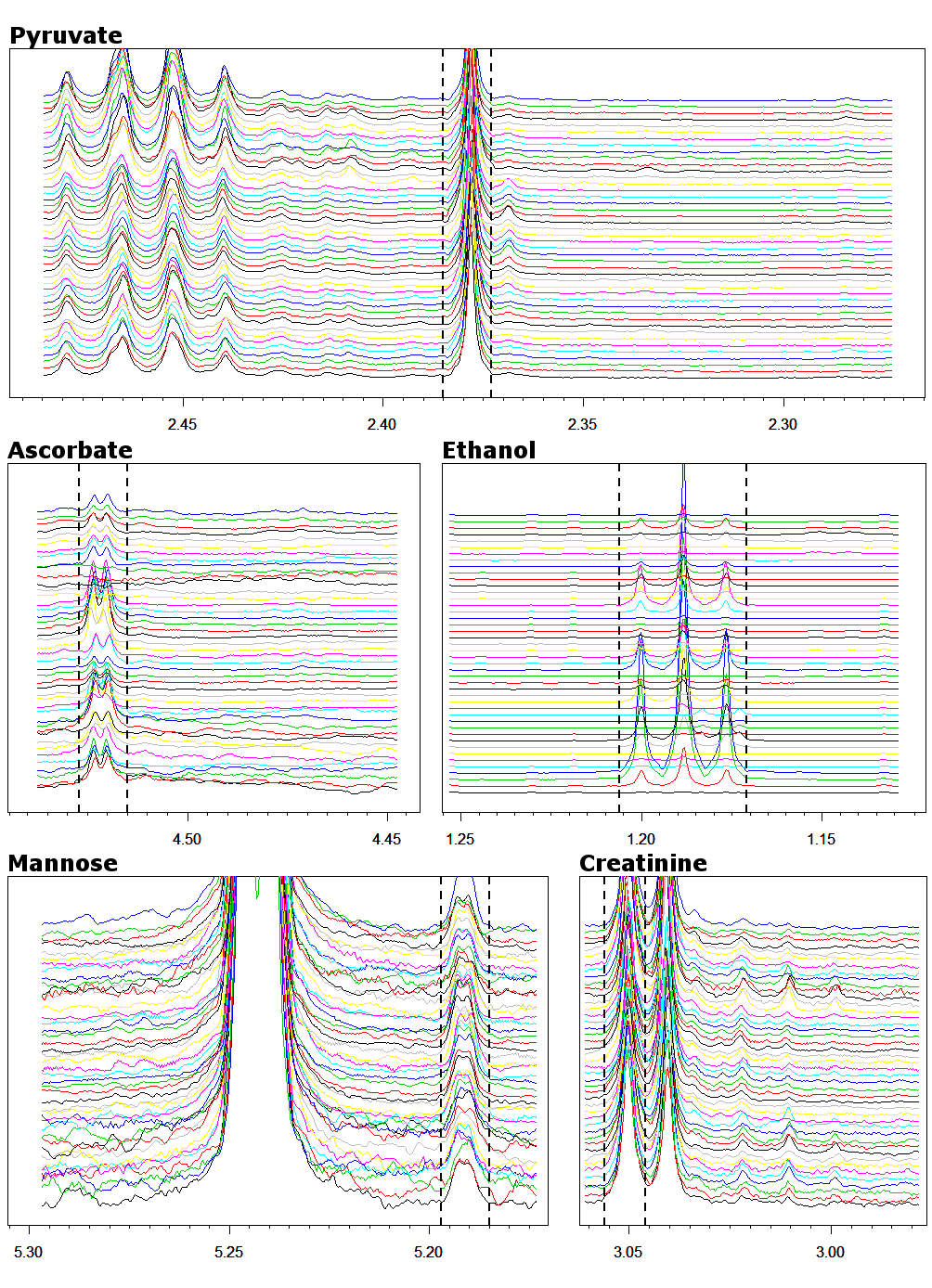


Figure continues from previous page


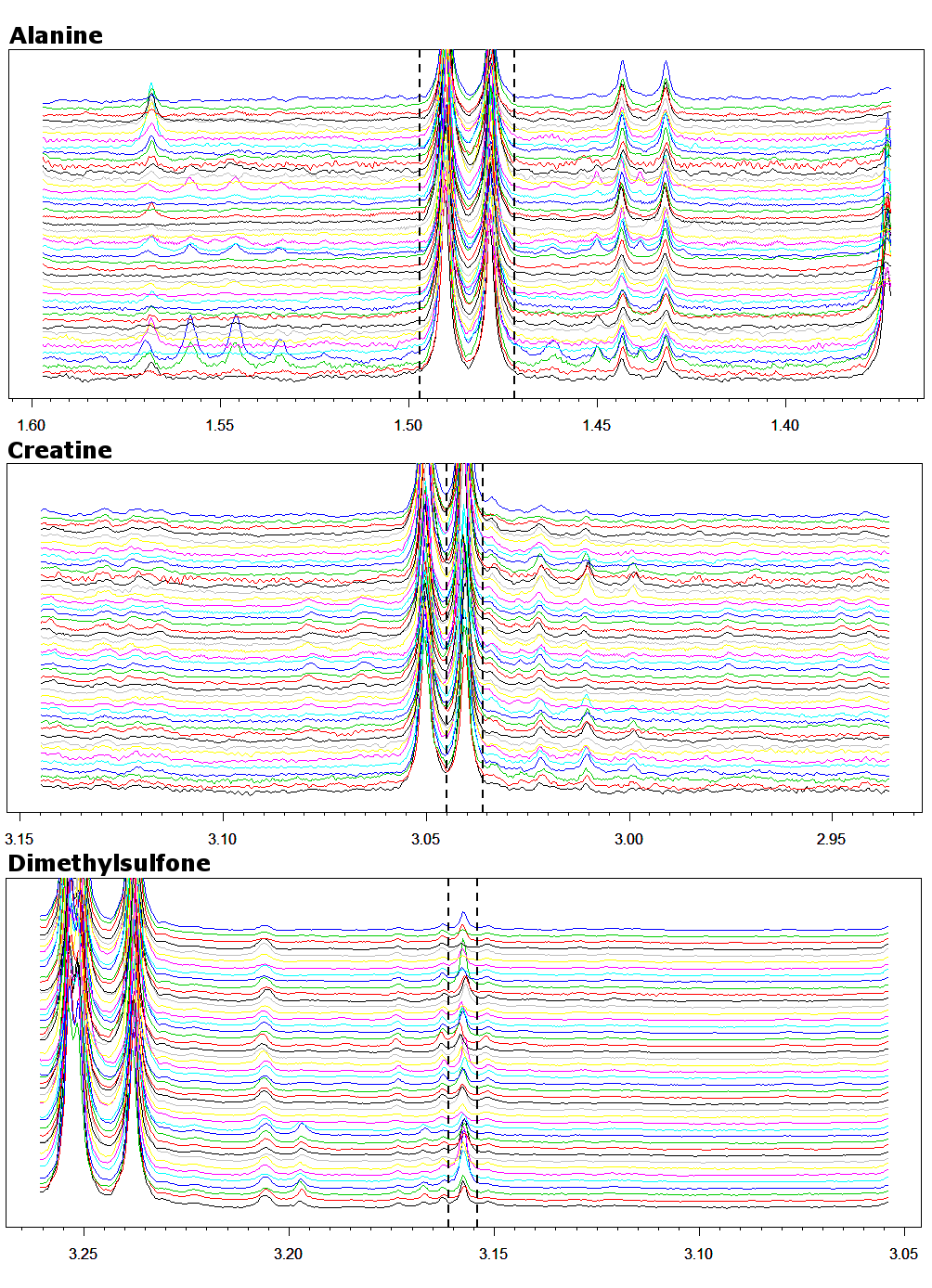


Figure continues from previous page


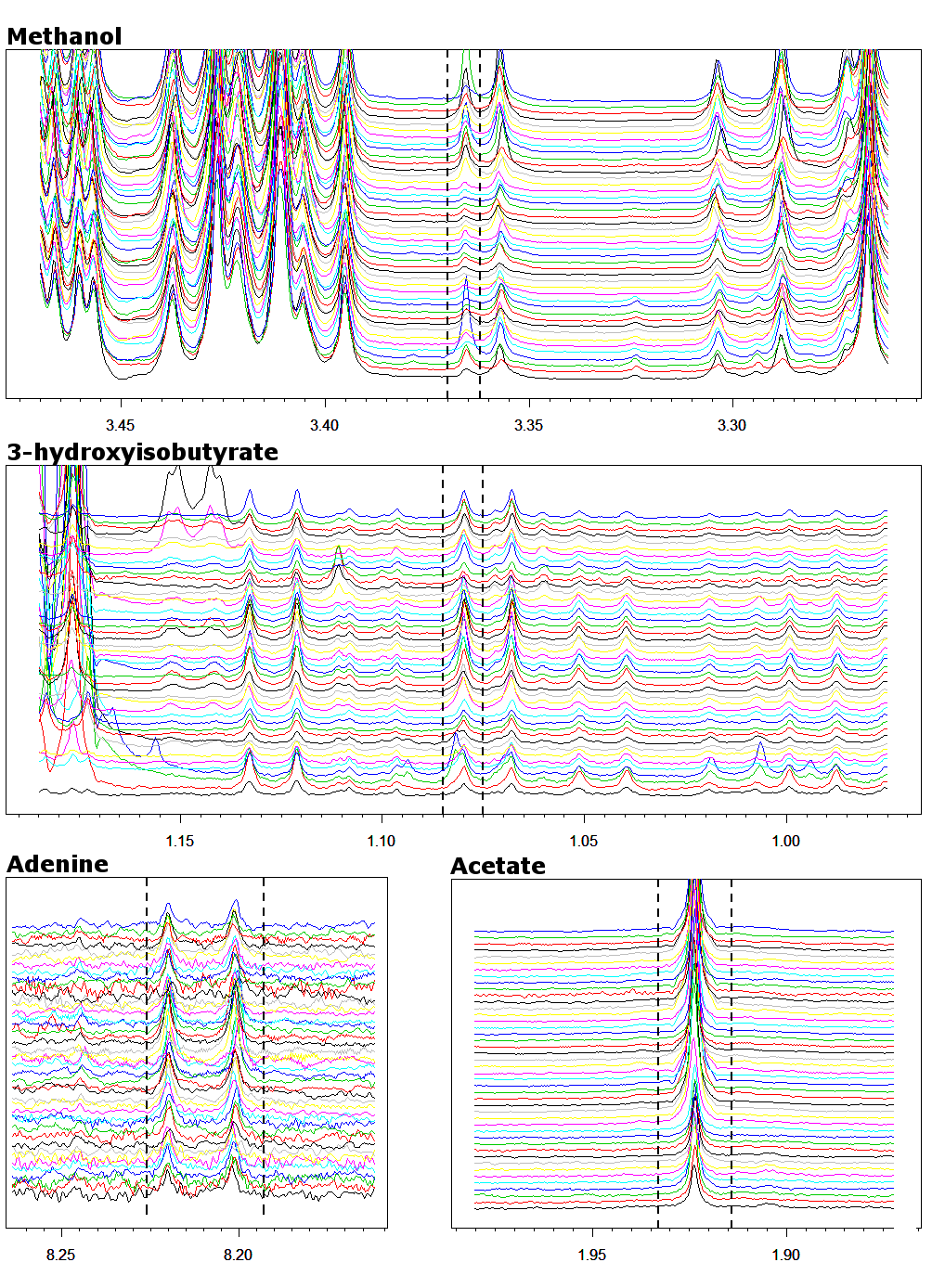


Figure continues from previous page


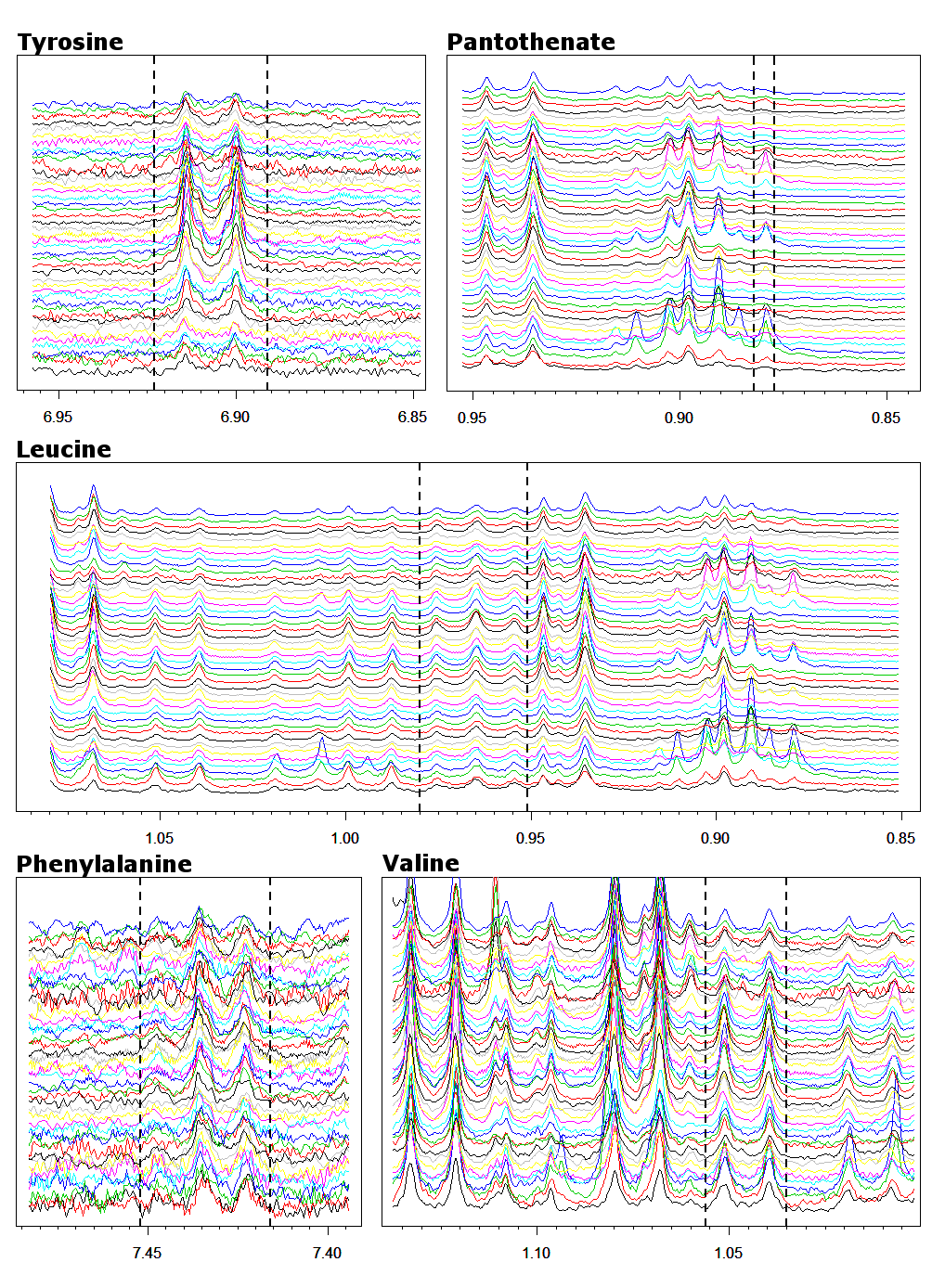


Figure continues from previous page


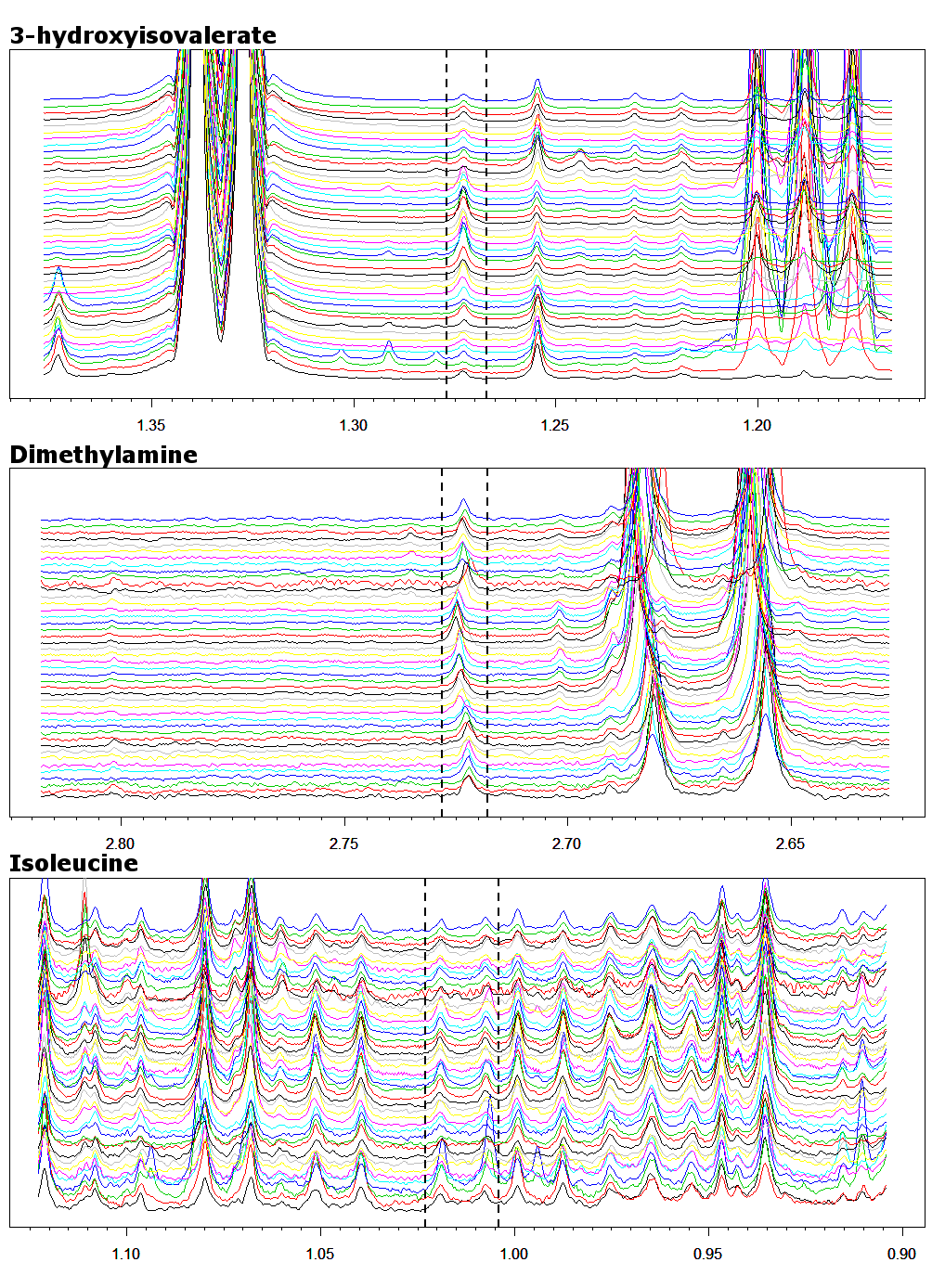


Figure continues from previous page


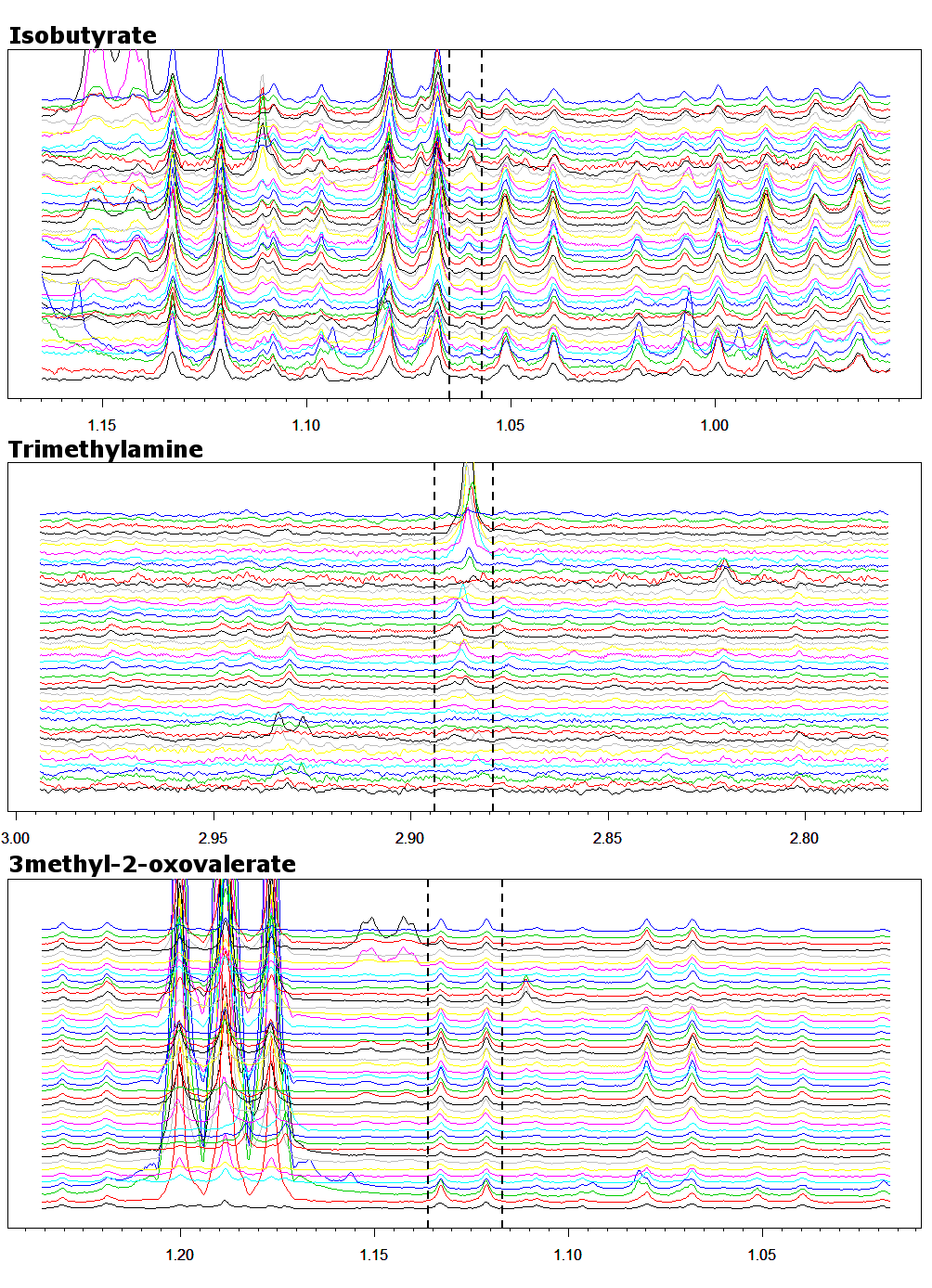


**Fig. b.** Pictorial representation of the assignment procedure of ^1^H-NMR signals performed through Chenomx suite (Chenomx Inc., Canada, ver 8.1) standard (ver. 10) and HMDB (ver. 2) data banks. For each molecule listed in table 1 of the main document, the panel with thick border shows the signal employed for quantification, while possible other panels show signals from the same molecule confirming the assignment. Each panel shows a convenient portion of the spectrum registered on the sample with the highest concentration of the molecule (black line), the calculated spectrum of the pure molecule (blue line), the point-by-point difference between registered and calculated spectrum. The triangle underneath each signal evidences, when blue, the plausibility of its chemical shift at the experimental pH.


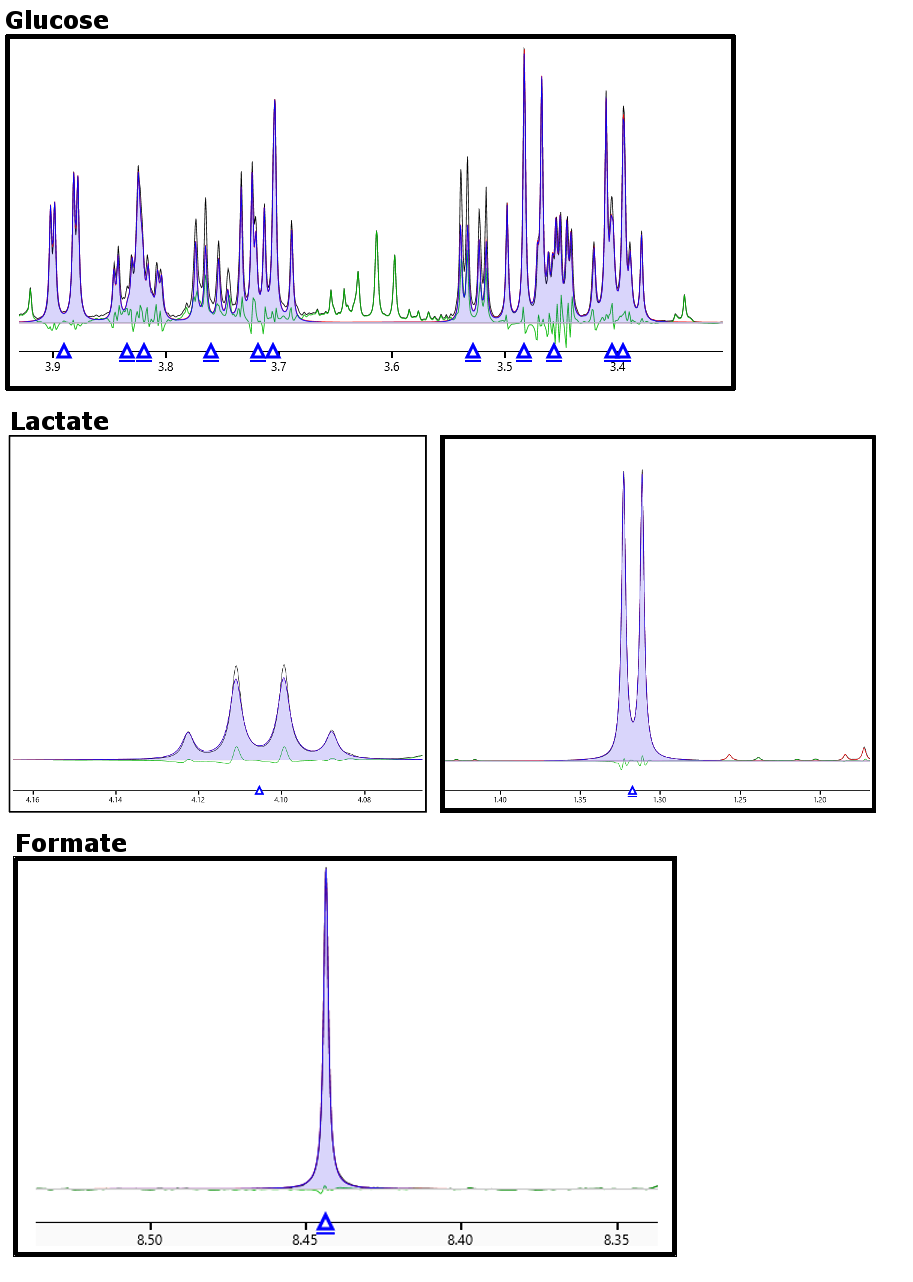


Figure continues from previous page


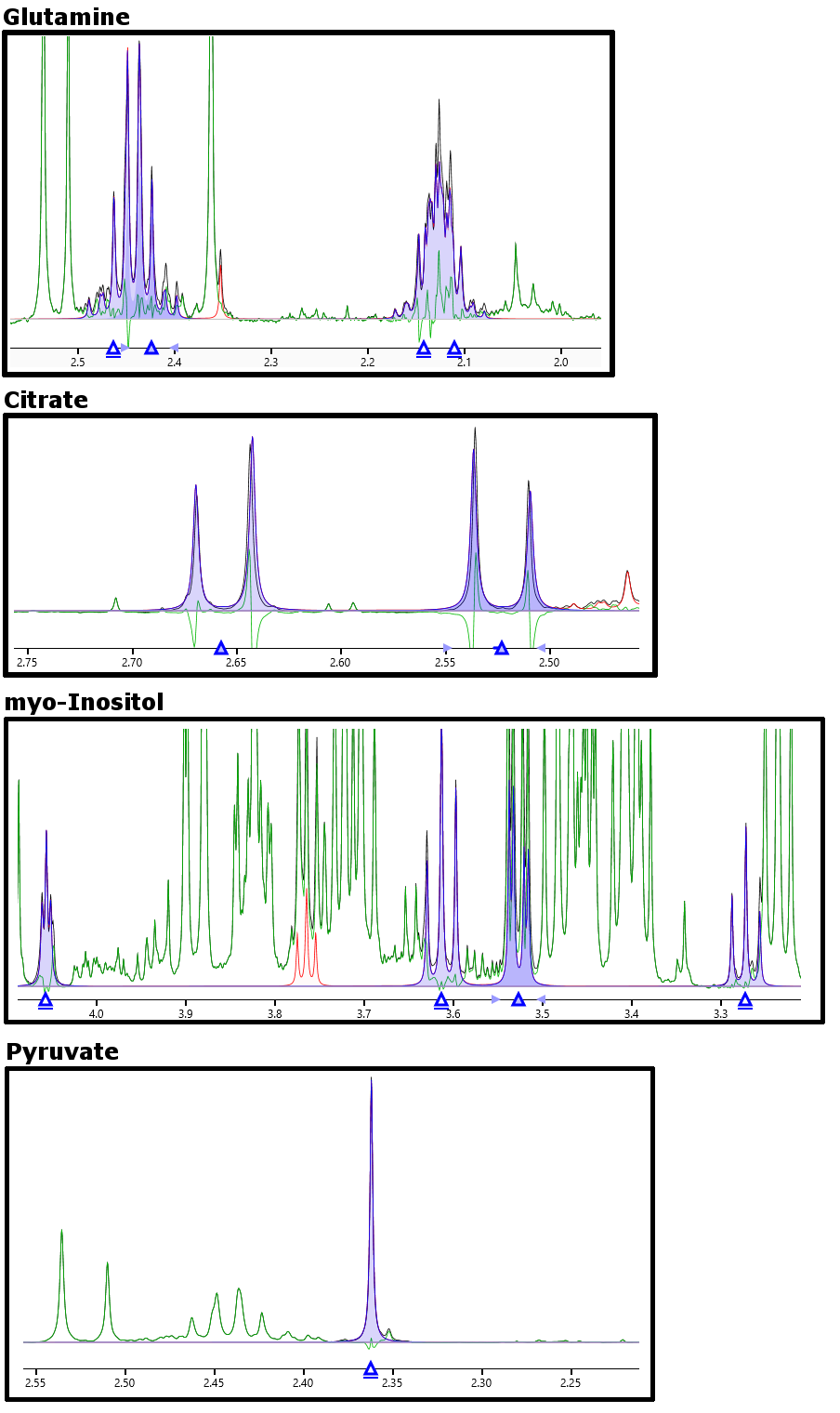


Figure continues from previous page


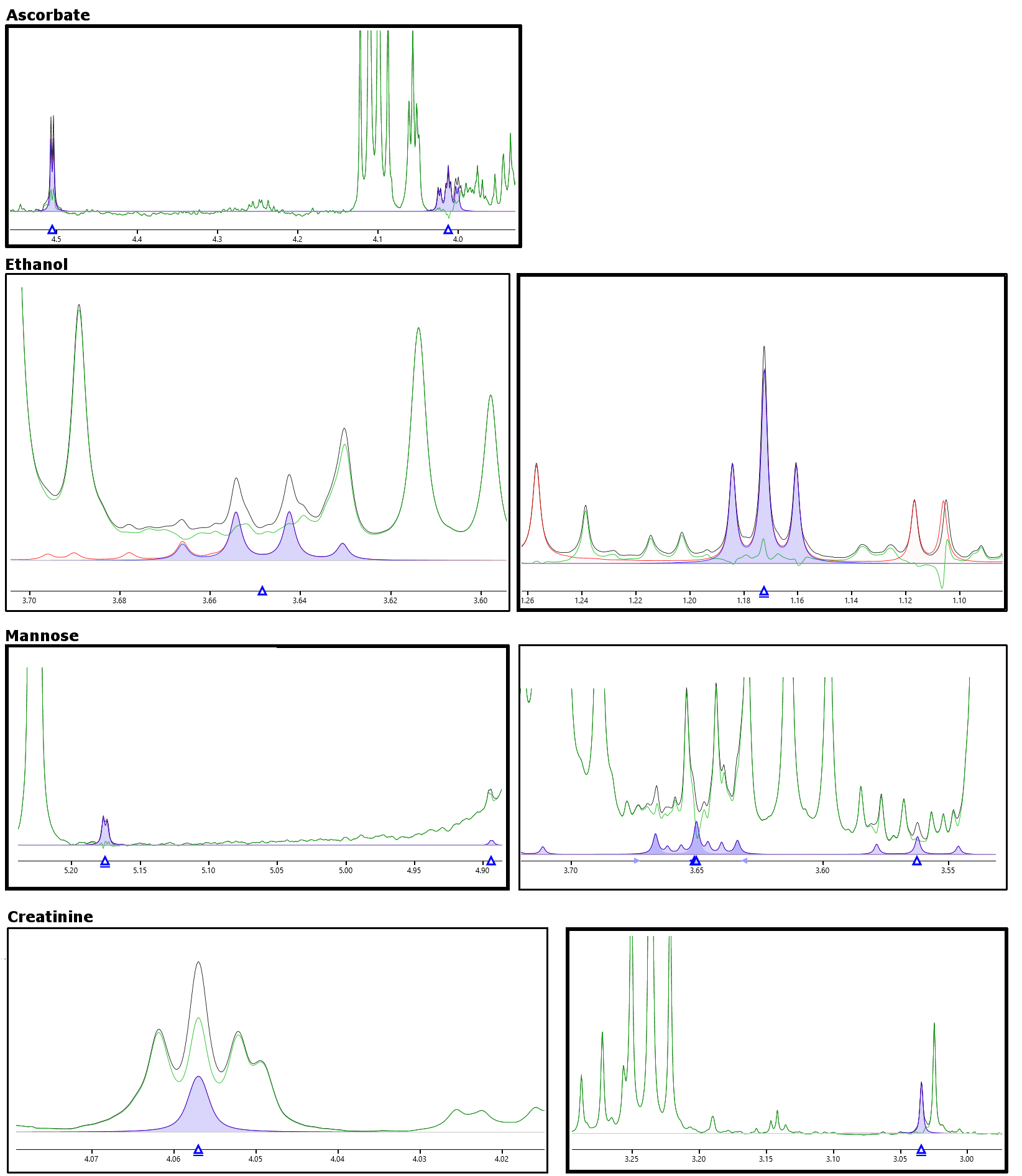


Figure continues from previous page


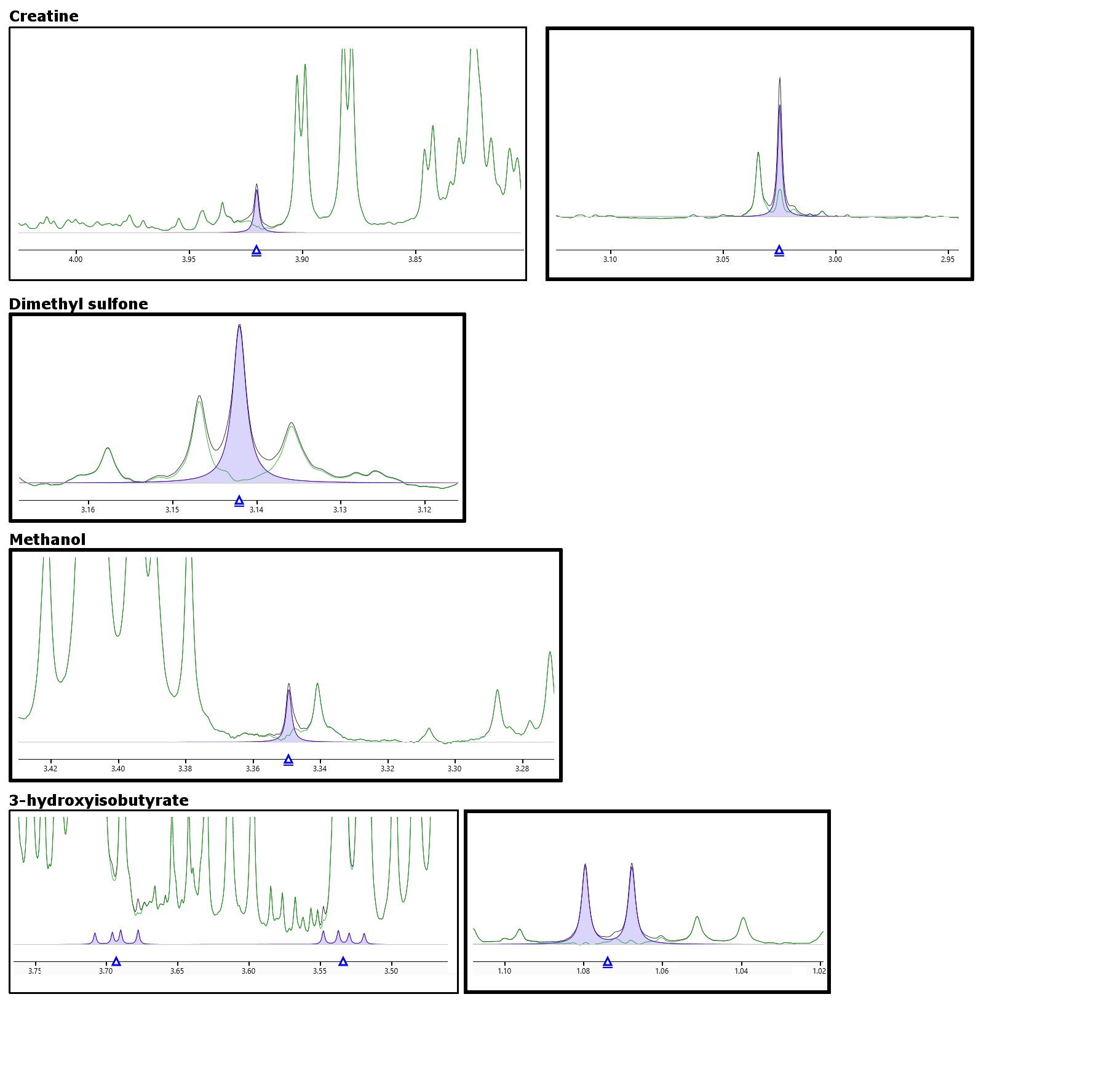


Figure continues from previous page


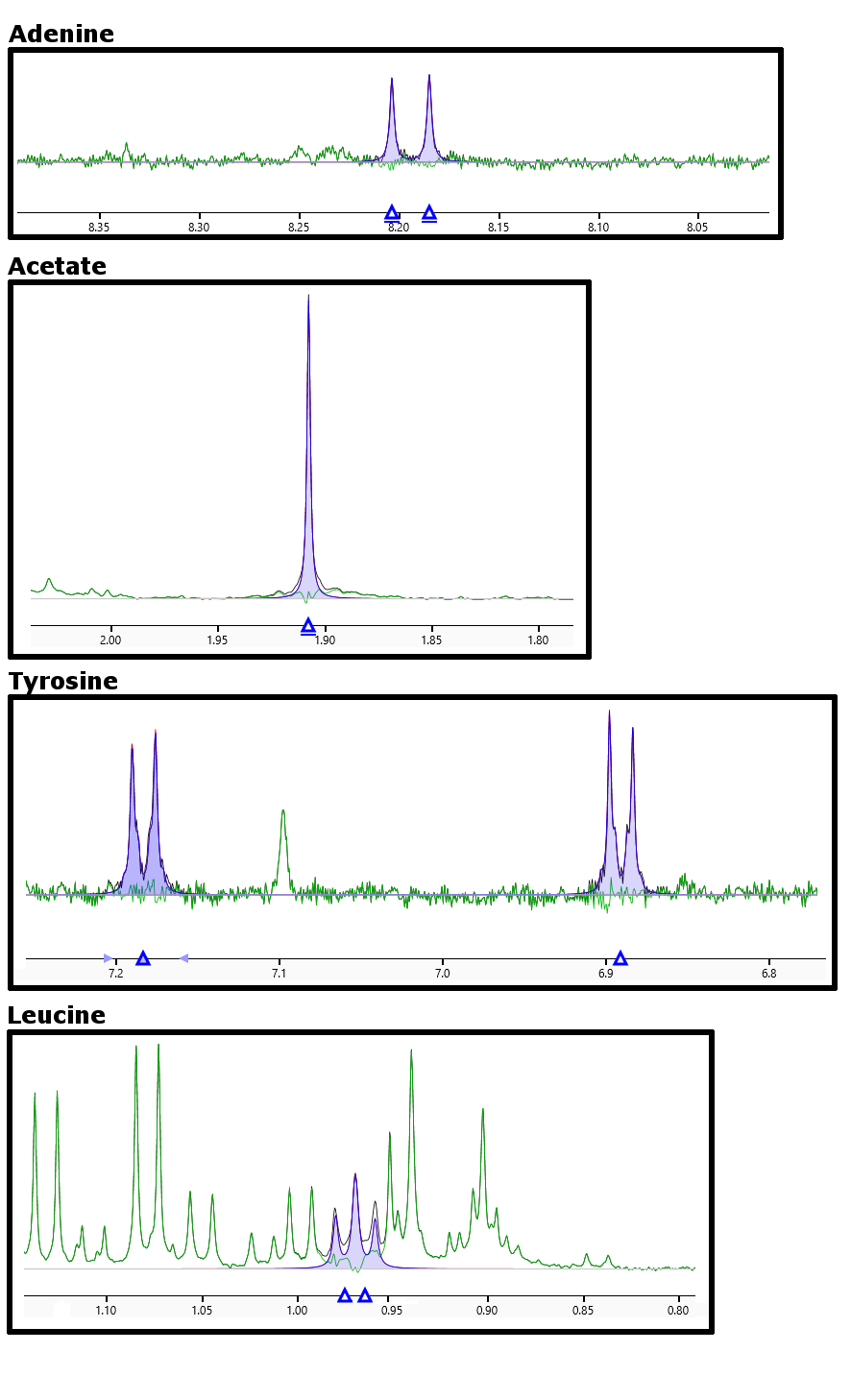


Figure continues from previous page


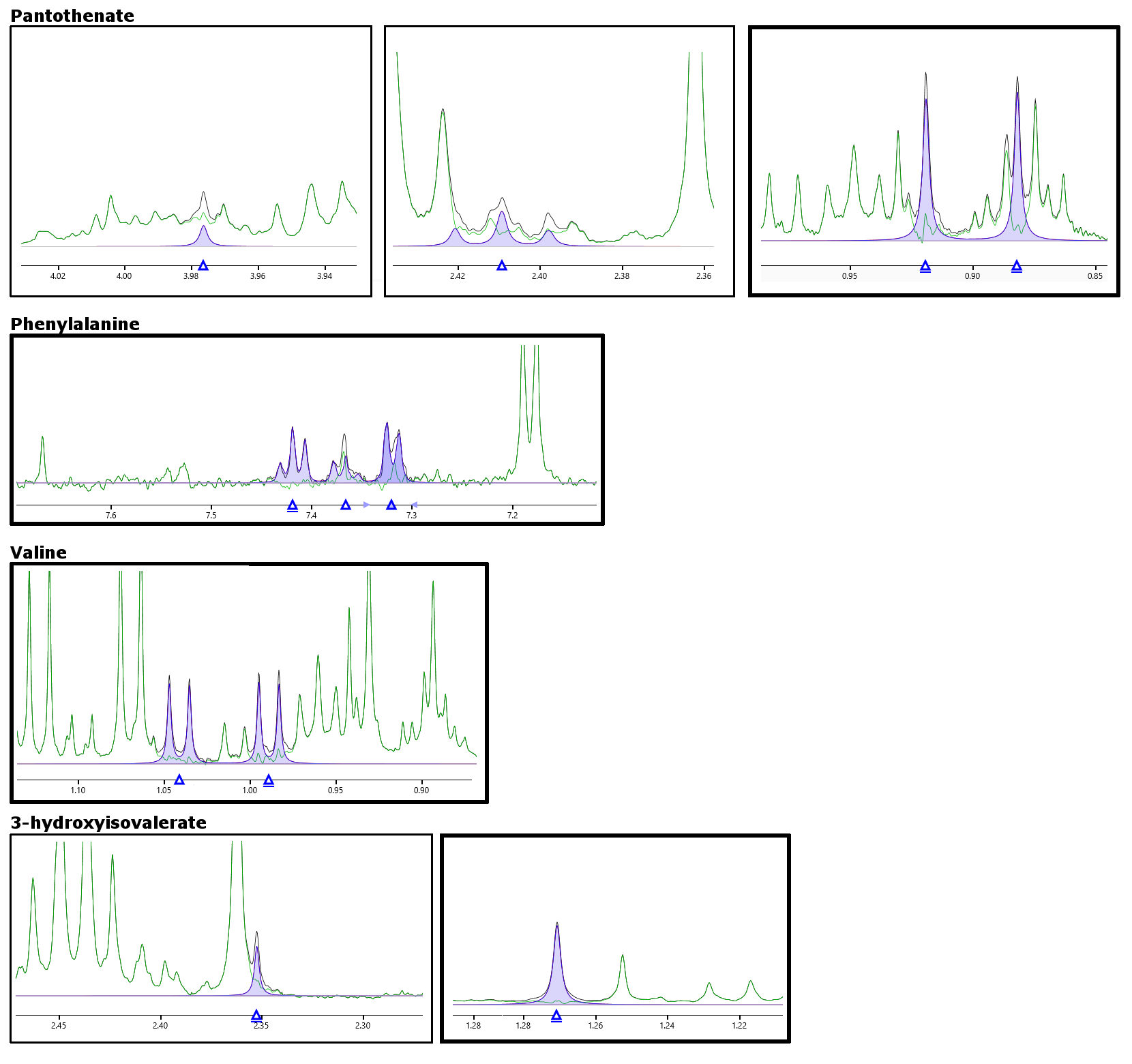


Figure continues from previous page


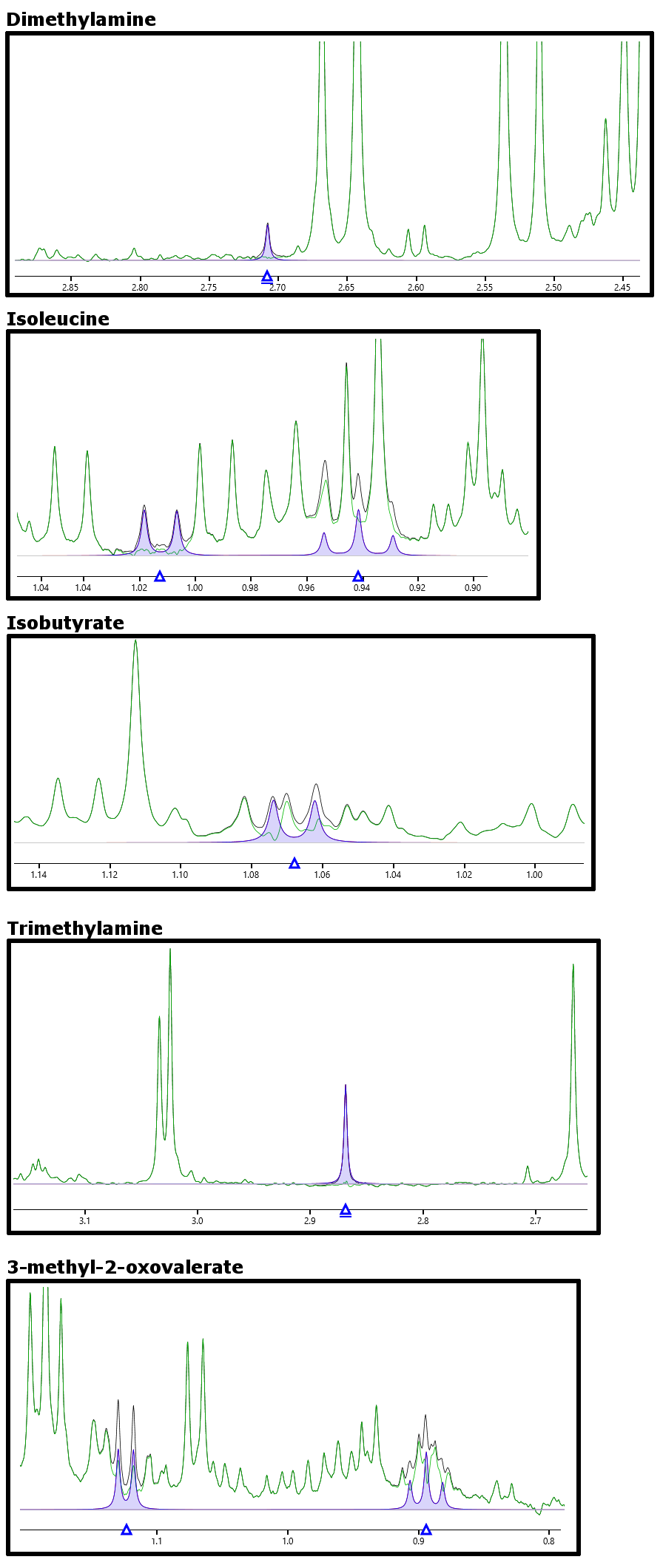


**Fig. c.** A) Portion of COSY spectrum, registered on a sample collected at day 50, showing the cross-peak between the signals in the 1.117 - 1.136 ppm range and a signal at 2.92 ppm, respectively in agreement with the methyl side chain group and the methine proton of 3-methyl-2-oxovalerate, shown in the insert. B) Portion of a JRES spectrum, registered on the same sample, showing that the signals in the 1.117 - 1.136 ppm range are part of a doublet, with a J of 7 Hz, in agreement with methyl side chain group of 3-methyl-2-oxovalerate.


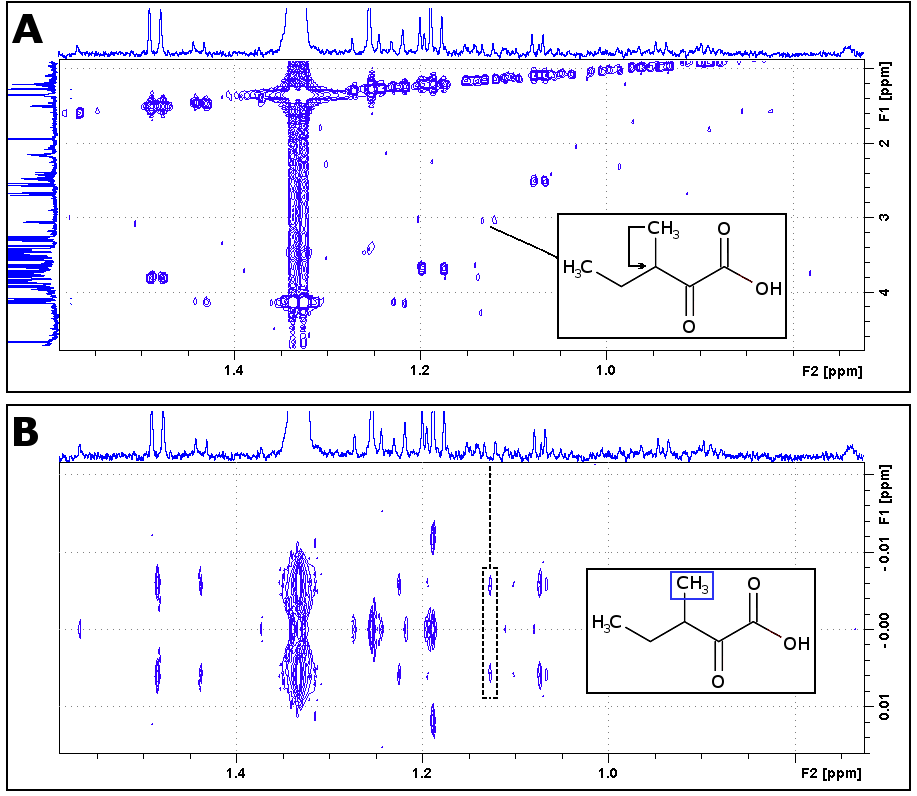

Supplement: S1 Fig — (DOCX) [file pone.0157623.s001.docx]
