# Supplementary material for: Age-Related 1H NMR Characterization of Cerebrospinal Fluid in Newborn and Young Healthy Piglets
Source: PLoS One. 2016 Jul 8;11(7):e0157623. doi: 10.1371/journal.pone.0157623 (PMC4938496; doi:10.1371/journal.pone.0157623)
Supplement: S1 File — (DOCX) [file pone.0157623.s002.docx]

# Sample Description

*Reference to biological sample provenance details*: Animals used in this study were Large White x Landrace x Duroc commercial hybrids. Pregnant sows (for P05 animals) and weaned piglets (for P30 and P50 animals) were delivered to our facility from Societa' Agricola Pasotti S.s, Imola 40026, Italy. All animals were enrolled as negative controls or as pre-treatment individuals in different protocols approved by the Italian Ministry of Health (art.7, D.Lgs 116/92). The sampling procedure was performed under general anaesthesia in order to avoid stress and guarantee the welfare of the animals. All pigs were constantly monitored during and after the procedure to rule out any possible complication. According to the individuals’ protocols, all animals were eventually euthanized upon intravenous administration of Tanax (0.3 ml/kg; MSD Animal health, Milano, Italy) after general anesthesia.

*pH of the sample after buffering*: 7.00 ± 0.02

*Field frequency lock*: D_2_O

*Additional solutes*: phosphate buffer at pH 7.00 (0.167M); sodium azide (0.33 mM)

*Solvents*: H_2_O/D_2_O (80/20 ratio)

*Chemical shift and concentration standard*: 3-(trimethylsilyl)-propionic-2,2,3,3-d4 acid sodium salt (TSP) (1.04 mM)

# Instrument Description

*Geographical location of the instrument*: Bologna University, P.za Goidanich 60, 47521 Cesena (Italy)

*Magnet*: manufacturer Bruker, model BZH 22/600/70F, sn z101339

*Probe*: manufacturer Bruker, model BBI 600 MHz 5mm, sn 2856801

*Console*: Bruker, Avance III 600, sn 0171

*Acquisition computer*: manufacturer HP, model z400 workstation, operating system windows 7 professional, applicalion software Topspin 3.0

*Autosampler*: manufacturer Bruker, B-ACS 60, application software Icon NMR, sn W3004463/00/0.00/0355

# Acquisition Parameters

*Sample details*: sample placed in tubes with internal diameter of 5 mm, sample temperature in autosampler 294 K, sample temperature in magnet 298K

*Instrument operation details*: sample not spinning, water signal presaturation during relaxation, pulse sequence T_2_ filter using Carr-Purcell-Meiboom-Gill sequence, pulse sequence file cpmgpr1d version 1.6.2.1 2009/12/14

*Data acquisition details*: number of steady state scans 16, number of scans 256, relaxation delay 5s, dwell time 69.6 μs, number of data points acquired 32K, o1 2822.79, acquisition nucleus ^1^H, average 90° pulse width of acquisition nucleus 10.8 μs, calculated for each sample

*Quality control*: TSP signal width at 5% of its total height.

# FID and Spectral Processing Parameters

*Time-based to frequency-based data transformation method*: Fourier transform with group delay compensation (PKNL), TSP to reference the spectrum, processing software Topspin 3.0

# Spectral Quantitation Parameter Set

*Quantitation type*: Integration of spectral regions by sum of spectral points, after probabilistic quotient normalization applied to the entire spectra array.

*Quantitation algorithm*: In-house built algorithm “peak area” version 26.02.2015, R computational language.

# Analysis Description

*Date and time of data acquisition*: from 05.11.2015 to 06.11.2015

*Institution*: Bologna University

*Operator and supervisor*: Luca Laghi
